# Model Free Approach to Kinetic Analysis of Real-Time Hyperpolarized ^13^C Magnetic Resonance Spectroscopy Data

**DOI:** 10.1371/journal.pone.0071996

**Published:** 2013-09-04

**Authors:** Deborah K. Hill, Matthew R. Orton, Erika Mariotti, Jessica K. R. Boult, Rafal Panek, Maysam Jafar, Harold G. Parkes, Yann Jamin, Maria Falck Miniotis, Nada M. S. Al-Saffar, Mounia Beloueche-Babari, Simon P. Robinson, Martin O. Leach, Yuen-Li Chung, Thomas R. Eykyn

**Affiliations:** 1 Cancer Research UK (CR-UK) and Engineering and Physical Sciences Research Council (EPSRC) Cancer Imaging Centre, Division of Radiotherapy and Imaging, The Institute of Cancer Research and Royal Marsden NHS Trust, Sutton, Surrey, United Kingdom; 2 Division of Imaging Sciences and Biomedical Engineering, Kings College London, St. Thomas Hospital, London, United Kingdom; The Norwegian University of Science and Technology (NTNU), Norway

## Abstract

Real-time detection of the rates of metabolic flux, or exchange rates of endogenous enzymatic reactions, is now feasible in biological systems using Dynamic Nuclear Polarization Magnetic Resonance. Derivation of reaction rate kinetics from this technique typically requires multi-compartmental modeling of dynamic data, and results are therefore model-dependent and prone to misinterpretation. We present a model-free formulism based on the ratio of total areas under the curve (AUC) of the injected and product metabolite, for example pyruvate and lactate. A theoretical framework to support this novel analysis approach is described, and demonstrates that the AUC ratio is proportional to the forward rate constant *k*. We show that the model-free approach strongly correlates with *k* for whole cell *in vitro* experiments across a range of cancer cell lines, and detects response in cells treated with the pan-class I PI3K inhibitor GDC-0941 with comparable or greater sensitivity. The same result is seen *in vivo* with tumor xenograft-bearing mice, in control tumors and following drug treatment with dichloroacetate. An important finding is that the area under the curve is independent of both the input function and of any other metabolic pathways arising from the injected metabolite. This model-free approach provides a robust and clinically relevant alternative to kinetic model-based rate measurements in the clinical translation of hyperpolarized ^13^C metabolic imaging in humans, where measurement of the input function can be problematic.

## Introduction

Magnetic resonance spectroscopy (MRS) is capable of distinguishing endogenous chemical metabolites together with xenobiotics in whole cells *in vitro*, in perfused organs *ex vivo* as well as providing non-invasive measurements *in vivo*. Compared with other imaging modalities, such as positron emission tomography (PET) or single photon emission computed tomography (SPECT), MRS has lower sensitivity, but its chemical specificity enables clinical identification of tumor types [1], steady state metabolomic investigations of health and disease [2] as well as treatment response to novel therapies and drug toxicology [3]–[5]. Dynamic Nuclear Polarization (DNP) provides a factor of 10^4^–10^5^ MRS signal enhancement [6], which allows for real-time metabolic imaging of enzyme kinetics in biological systems using ^13^C MRS. The most widely explored application of hyperpolarized ^13^C MRS to date is the measurement of apparent reaction rate constants governing pyruvate-lactate exchange, which reflect lactate dehydrogenase (LDH) activity. LDH is a key metabolic enzyme that is commonly upregulated in cancer, and is central to the altered energy metabolism evident in cancer, known as the Warburg effect [7]. Therapy-induced changes in the forward apparent reaction rate constant have been identified as a potential early metabolic biomarker of treatment response to inhibitors of cell signaling such as phosphoinositide 3-kinase (PI3K) [8], and to chemotherapy treatment, which induced apoptosis [9].

Following injection of hyperpolarized [1-^13^C]pyruvate into either a suspension of cells *in vitro*, or via intravenous injection *in vivo*, [1-^13^C]pyruvate is transported into cells via monocarboxylate 1 (MCT1) transporters. Inside the cell, [1-^13^C]pyruvate is predominantly in fast exchange with intracellular lactate, resulting in the formation of [1-^13^C]lactate, which is mainly transported out of the cell by MCT4. LDH catalyzes the interconversion of pyruvate and lactate, with concomitant interconversion of its cofactors, NADH and NAD^+^. [1-^13^C]pyruvate can also be shuttled into the mitochondria and metabolized in the TCA cycle (demonstrated by the formation of [1-^13^C]bicarbonate, which is in equilibrium with carbon dioxide, under the action of the enzyme carbonic anhydrase (CA)), or converted to [1-^13^C]alanine in the cytosol by alanine transaminase (ALT) [10]. These apparent reaction rate constants are typically determined by kinetic modeling of the dynamic data using the McConnell equations (modified Bloch equations) [11].

Kinetic modeling of DNP dynamic ^13^C data requires prior knowledge of the reaction mechanism including all of the generated metabolites, and for *in vivo* measurements an accurate estimate of the arterial input function (AIF) is also required. It has been shown recently that errors in the AIF critically influence estimates of the apparent rate constants from kinetic modeling [12]. A number of strategies for measuring the AIF have been proposed, including the addition of unreactive substrates (e.g. urea) [13] and signal localization on a suitable vessel [12], but these methods involve more complex experimental or acquisition designs that will, in practice, decrease the overall robustness of the technique. Alternative modeling approaches that do not require measurement of the AIF have been investigated *in vivo*. In one method, a piecewise fitting approach is implemented, which separates out the influence of the AIF from the metabolic conversion [14], [15]. Another approach neglects in- and out-flow effects of the generated metabolite, assuming them to be equal, and approximates the differential equations by difference equations. This approach removes the influence of the AIF [16].

**Figure 1:**
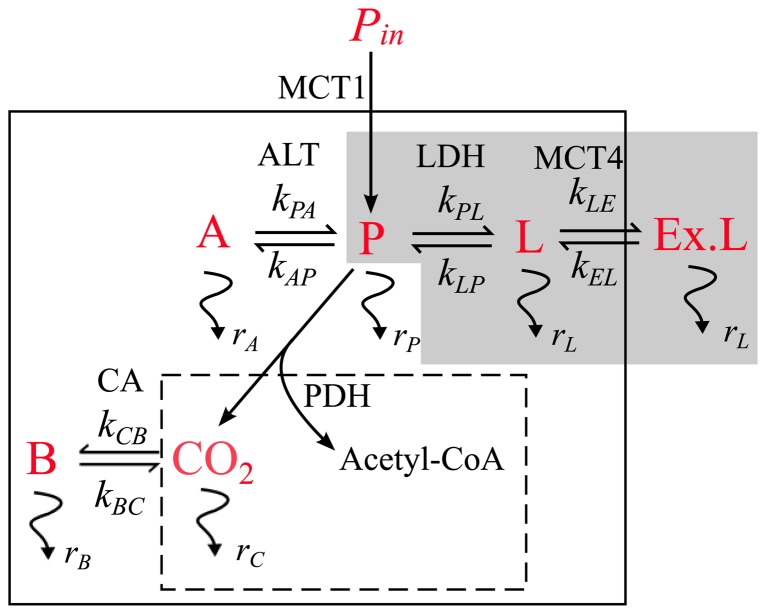
A representation of the fate of hyperpolarized [1-^13^C]pyruvate (P) that is injected into a system with input function *P_in_*(*t*). Observable ^13^C signals originating from [1-^13^C]pyruvate are indicated in red. The schematic shows the transport of pyruvate into a cell, facilitated by MCT1 transporters, and its conversion to other metabolites. Solid lines correspond to the cell membrane and dashed lines to the mitochondrial membrane. 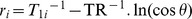 is the effective relaxation rate of the hyperpolarized signal for metabolite *i*. Conversion to metabolites [1-^13^C]lactate (L), [1-^13^C]alanine (A), and [1-^13^C]bicarbonate (B) occur with reaction rates (*k*), and enzymes that catalyze reactions are shown. *k_EL_* and *k_LE_* are the rates of lactate transport into and out of the cell, governed by the MCT4 transporters. Entry of pyruvate into the TCA cycle results in conversion of the 1-^13^C label to CO_2_ and then to bicarbonate. Acetyl-CoA is not seen owing to the [1-^13^C] label of pyruvate being utilized in the formation of CO_2_. The grey box indicates the terms that need to be considered for the AUC ratio analysis method when the reaction of interest is pyruvate-lactate conversion, whereas kinetic modeling requires fitting of all terms depicted here, except for acetyl-CoA.

Here, we investigate the potential of a simpler, model-free analysis method to quantitatively characterize apparent hyperpolarized reaction kinetics by measuring the ratio of the total areas under the dynamic curves (AUC) of the generated metabolite and injected metabolite. The purpose of this paper is to derive formulae for the AUC ratio for a number of different models where we show that this ratio is proportional to the forward apparent rate constant. From this analysis we demonstrate that the method is independent of the AIF, and is applicable for reactions where multiple metabolites are generated, for example *in vivo* tumor, cardiac and hepatic studies. We present a comparison of the AUC ratio method with *k_PL_* values derived from both 2-site and 3-site kinetic models for *in vitro* data performed across a wide variety of cancer cell types (CHL-1, HCT116 Bax-KO, HT29, SF188, SW1222, WM266-4, PC3) and also for PC3 prostate cancer cells in response to the PI3K inhibitor, GDC-0941. We also apply the AUC analysis methods to *in vivo* data sets from mice bearing subcutaneous HT29 or SW1222 colon cancer xenografts treated with the pyruvate dehydrogenase kinase (PDK) inhibitor dichloroacetate (DCA). DCA acts as an anti-cancer agent by inhibiting PDK, which prevents inactivation of PDH, and as a result, pyruvate decarboxylation in the mitochondria is facilitated, forming acetyl-coA (Fig. 1). DCA has been shown to induce apoptosis, decrease proliferation and inhibit tumor growth, without apparent toxicity [17]. Therefore, response to DCA treatment would be characterized by a drop in *k_PL_* or by a drop in AUC ratio compared with controls.

Following the recent phase I clinical trial of hyperpolarized pyruvate in prostate cancer at UCSF, San Francisco, CA, there is increasing potential for the clinical application of hyperpolarized ^13^C NMR. This simpler AUC method is an attractive and more clinically applicable alternative to kinetic modeling for the quantification of apparent hyperpolarized kinetics.

## Theory

The metabolic fate of hyperpolarized [1-^13^C]pyruvate can be monitored in real-time by MRS, and apparent reaction rates of LDH can be derived from kinetic modeling of the system. Figure 1 shows a schematic of the different metabolic fates of pyruvate in a cell, where MR observable ^13^C signals generated from the metabolism of [1-^13^C]pyruvate are depicted in red. The MRS signals are influenced by hyperpolarized signal relaxation rates, the input function of the injected metabolite, conversion to other metabolites and transport between intracellular and extracellular compartments, as well as loss of polarization due to application of the radio-frequency (RF) pulse. The most commonly used models in the literature are 2-site exchange [9], [14], [18] and 3-site exchange [19], which are based on the modified Bloch equations [11]. The 2-site model does not take cellular transport into account, and therefore assumes that all hyperpolarized signals are intracellular, whereas the 3-site exchange model differentiates between intracellular and extracellular lactate, thus taking lactate efflux into account. In this study we have compared the simpler AUC ratio method with effective rate constant estimates derived from both 2-site and 3-site kinetic models for *in vitro* data, and from a kinetic model for *in vivo* data, which also includes (intra-cellular) alanine.

Equations (1–2) are the modified Bloch equations, which describe pyruvate-lactate exchange rates, where it is assumed that the exchange reaction obeys first order kinetics.
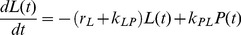
(1)

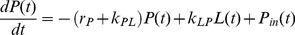
(2)where 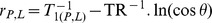 denote the effective relaxation rates of hyperpolarized ^13^C signals of pyruvate (*P*) and lactate (*L*) respectively (accounting for signal loss due to RF excitations of angle *θ* with repetition time TR, see Text S1), *k_PL_* and *k_LP_* are the effective rate constants for the chemical exchange reaction and *P_in_*(*t*) is the pyruvate input curve. Manipulation of Eq. (1) using Laplace transforms (described in Text S1) yields an elegant expression for the AUC ratio, as follows:
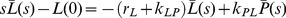
(3)Where s is the Laplace variable. Since there is no hyperpolarized lactate at time t = 0 it follows that *L*(0) = 0 and therefore,
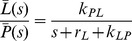
(4)For a general signal *X*(*t*) the Laplace transform has the property
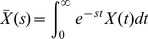
(5a)

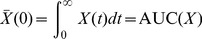
(5b)Within a finite time the hyperpolarized signal relaxes to its equilibrium value, which is undetectable in these experiments and therefore,
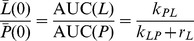
(6)
Eq. (6) demonstrates that the ratio of lactate AUC to pyruvate AUC is proportional to *k_PL_*. This result is derived from equation (1) alone, which has two remarkable, and somewhat counterintuitive, consequences. First, that it is independent of the pyruvate input curve, and second that it is also independent of both the pyruvate relaxation rate and rate constants associated with conversion of pyruvate to any other metabolites, for example alanine, since these only appear in the pyruvate equation (Eq. (2)).

Since DNP data are sampled at discrete time-points, the AUC integral in equation (5b) is approximated with a sum, and the AUC ratio is computed using:
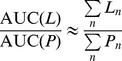
(7)In practice this approximation is acceptable since the sampling rate (around 2 seconds) is an order of magnitude faster than even the most transient features of the metabolite curves.

A similar equation can be derived for the 3-site model, which describes the relationship between the AUC ratio and *k_PL_*, and accounts for lactate transport (Eq. (8)). This model assumes that pyruvate transport into the cell via monocarboxylate transporter 1 (MCT1) occurs very quickly, and is approximated to an instantaneous process in the model, whereas lactate efflux via MCT4 occurs more slowly, and is included as a measurable process in the model. This assumption is based on the relative affinities of the transporters for pyruvate and lactate respectively [20]. For the derivation of the 3-site model see Text S1, ‘Three-site plus-one Model’.
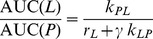
(8)Where *γ* = (*k_EL_*+*r_L_*)(*k_LE_*+*k_EL_*+*r_L_*)^−1^, and *k_LE_* and *k_EL_* are respectively the rates of lactate transport out of and into cells (Fig. 1). The 2-site model assumes that there is no lactate transport into or out of the cell, so *k_LE_* = *k_EL_* = 0 giving *γ* = 1 and Eq. (8) reverts to Eq (6) as expected.

For a simple one way reaction with first order kinetics, (i.e. pyruvate is converted to lactate with no reverse flux) the analysis is also valid, since in this case *k_LP_* = 0 and the AUC ratio simplifies to *k_PL_*/*r_L_* for the 2- and 3-site models. Both the 2- and 3-site analyses demonstrate that the AUC ratio is proportional to the forward rate constant, but to be a useful metric we require that, in practice, the denominator term have sufficiently low variation relative to the variation in *k_PL_*. We demonstrate this empirically by fitting *in vitro* and *in vivo* hyperpolarized data with kinetic models to obtain *k_PL_* values, which are compared with AUC measurements obtained from the same data sets according to Equation (7).

Normalization of *in vitro* and *in vivo* data is performed differently; *k_PL_* values obtained directly from the kinetic model have units of /s, and *in vitro* data are often normalized to initial pyruvate concentration and total cell number (typically per million cells), yielding units of (for example) nmol/s/10^6^ cells. Similar normalization methods are more complicated *in vivo* owing to the distribution of hyperpolarized substrate throughout the vasculature of the animal, making it difficult to determine metabolite concentrations, and for the purpose of this paper *in vivo k_PL_* values are in units of /s.

## Methods

### Cell Preparation for MRS

All cells were cultured at 37°C in a humidified atmosphere containing 5% CO_2_. Cell culture medium had the addition of 10% fetal bovine serum (Invitrogen, UK) and (unless stated otherwise) 1% penicillin & streptomycin. **Cell lines**: CHL-1 melanoma (from American Type Culture Collection, ATCC, cultured in Dulbecco's Modified Eagle Medium (DMEM) containing glutamine and 1% non-essential amino acids), HCT116 Bax-KO colon carcinoma (a kind gift from Dr. Bert Vogelstein, Johns Hopkins Medical Center, USA; via Dr. Paul Clarke, ICR, Sutton, UK [21], cultured in DMEM with glutamine and 1% non-essential amino acids (Invitrogen, UK)), HT29 colon carcinoma (from American Type Culture Collection, ATCC, cultured in McCoy 5A Medium with glutamine and HEPES (Invitrogen, UK)), PC3 prostate adenocarcinoma (from American Type Culture Collection, ATCC, cultured in DMEM with glutamine (Invitrogen, UK)), pediatric SF188 glioblastoma (a kind gift from Dr. Daphne Haas-Kogan, UCSF, San Francisco, CA, USA [22], cultured in DMEM/F12 Ham's medium (Invitrogen, UK), without penicillin & streptomycin), SW1222 colon carcinoma (from The European Collection of Cell Cultures, ECACC, cultured in DMEM with glutamine (Invitrogen, UK)), WM266-4 melanoma (from American Type Culture Collection, ATCC, cultured in DMEM containing glutamine and 1% non-essential amino acids). PC3 cells were treated with a pan-class I PI3K inhibitor, GDC-0941, at 5× GI50 (5 µM) for 24 hours. To harvest cells, the floating fraction was discarded and adherent cells were rinsed twice with phosphate buffered saline (PBS). Cells were trypsinized and centrifuged for five minutes at 190 g. Cell number and viability were determined by haemocytometer and trypan blue staining. Cell pellets were immediately resuspended in 500 µl serum-free medium and MRS studies were carried out within 10 minutes of cell harvesting.

### In vitro MRS

MRS was performed at 37°C on a Bruker 11.7 T spectrometer. **Hyperpolarized ^13^C** ***in vitro***: 18 mg [1-^13^C]pyruvic acid (99% isotopically enriched, Sigma-Aldrich, UK containing 15 mM trityl free radical OX63, Oxford Instruments, UK) was polarized in a HyperSense® DNP polarizer (Oxford Instruments Molecular Biotools Ltd, UK) for 1 hour. The polarized sample was dissolved in 4 ml aqueous buffer (50 mM sodium lactate, 50 mM NaOH, 1 mM EDTA) resulting in a 50 mM pyruvate solution at pH 7, 37°C. A solution of 100 µl, 50 mM hyperpolarized [1-^13^C]pyruvate was mixed with 500 µl cell suspension, and ^13^C spectra were acquired every 2 s using a single scan and a 10° flip angle.

### Ethics Statement

All procedures performed on mice were approved by the Institute of Cancer Research Ethical Review Committee and with the authority of Personal and Project Licences issued by the UK Home Office under the Animals (Scientific Procedures) Act 1986, and in accordance with the United Kingdom National Cancer Research Institute guidelines for the welfare of animals in cancer research [23]. Tumour propagation and MRS were performed under general anaesthesia and every effort was made to minimise suffering at all times.

### In vivo MRS

MRS was performed on either a Bruker 7 T horizontal bore micro-imaging system or a Philips 3 T clinical scanner. ***In vivo*** **tumor implantation and DCA treatment:** Human HT29 carcinoma or SW1222 colon carcinoma cells (5×10^6^) were propagated subcutaneously in NCr nude mice. Tumors were scanned on day one, mice were then treated on days two and three with 200 mg/kg DCA p.o. and a final dose was given on day four, one hour before the post-treatment scan. **MR Coils:** Mice bearing cancer xenografts of volume 250–300 mm^3^ were positioned with their tumor within either a custom made 1.8 cm diameter (Bruker 7 T) or 2 cm diameter (Philips 3 T) ^13^C transmit/receive surface coil at the isocentre of the spectrometer. **Hyperpolarized ^13^C** ***in vivo*** **:** A solution weighing 26 mg [1-^13^C]pyruvic acid (99% isotopically enriched, Sigma Aldrich, United Kingdom) containing 15 mM trityl free radical OX63 (GE Healthcare) and 1.5 mM gadolinium Dotarem-DOTA (Guerbet, United Kingdom) was polarized in a HyperSense® DNP polarizer (Oxford Instruments Molecular Biotools Ltd, UK) for 1 hour. The hyperpolarized pyruvic acid was dissolved in 4 ml Trizma buffer containing 80 mM NaOH, 1 mM EDTA, 50 mM NaCl resulting in a 80 mM pyruvate solution at pH 7, 37°C. A solution of 175 µl 80 mM hyperpolarized [1-^13^C]pyruvate was administered *in situ* via a lateral tail vein over approximately 5 s. A series of 128 ^13^C spectra were recorded at 75 MHz (Bruker 7 T), every 2 s using a 20° pulse-and-acquire sequence (1 transient, 32 k time domain points, 15 kHz spectral width) or at 32 MHz (Philips 3 T) every 3 s using a 20° slice selective pulse-and-acquire sequence (10 mm slice thickness, 1 transient, 2048 time domain points, 8 kHz spectral width).

### Data analysis

Spectra were phase and baseline corrected, and peaks of interest selected and integrated over the time course of the experiment. **Kinetic Modeling:** Peaks were quantified using the Amares fitting tool in jMRUI (Philips 3 T spectra) or in TopSpin (Bruker 11.7 T and Bruker 7 T spectra). Kinetic modeling was carried out in Matlab (Mathworks®, UK) by fitting to the time series of peak areas. These time series were modeled with the modified Bloch equations and fitted using maximum likelihood estimation. A raised cosine input function was used to model the pyruvate bolus *in vivo*. A modified cost function was developed to optimize the fitting of *in vitro* data, which significantly improved the fit quality when compared to a least-squares approach (see Text S1, for more details). **AUC Ratio:** The time courses of integrals from hyperpolarized lactate and pyruvate signals were summed and the ratios of the total lactate/total pyruvate curves were calculated.

## Results

**Figure 2:**
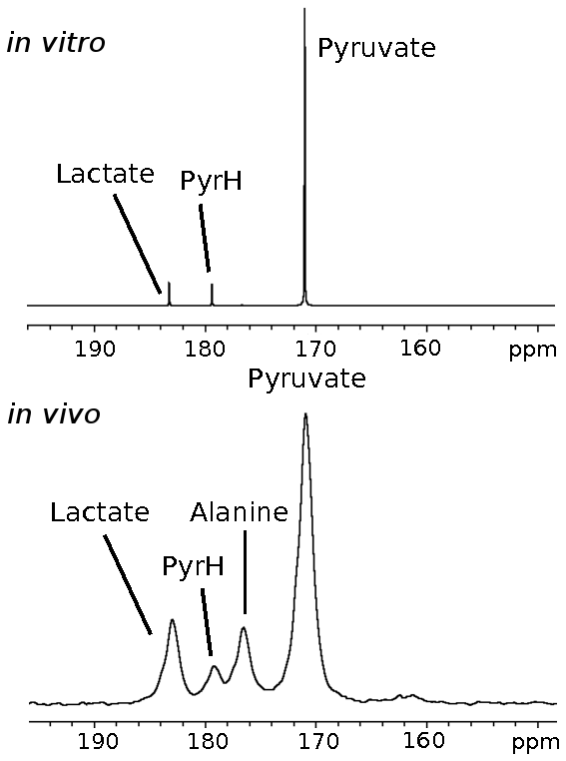
The sum of 128 dynamic ^13^C spectra from representative *in vitro* (top) and *in vivo* (bottom) data acquired after injection of hyperpolarized [1-^13^C]pyruvate. I*n vitro* spectra were acquired at 11.7 T from an SW1222 colon carcinoma cell suspension. Peaks arising from pyruvate (171 ppm), lactate (183 ppm) and pyruvate hydrate (179 ppm), an unreactive molecule formed from hydration of pyruvate. A very small peak at 177 ppm, attributed to alanine, was only observable once spectra were summed. *In vivo* spectra were acquired at 7 T from a control HT29 colon carcinoma xenograft and shows peaks arising from pyruvate, lactate, pyruvate hydrate and alanine.

Example spectra from *in vitro* and *in vivo* data sets are shown in Figure 2, where all 128 dynamic spectra were summed. The *in vitro* spectra show peaks arising from the parent pyruvate, from lactate and from pyruvate hydrate, an unreactive molecule formed from hydration of pyruvate. A very small peak at 177 ppm, attributed to alanine, was only observable once spectra were summed. No other significant metabolites were observed in any of the cell data. The *in vivo* spectrum shows peaks arising from the parent pyruvate, lactate, pyruvate hydrate and alanine.

**Figure 3:**
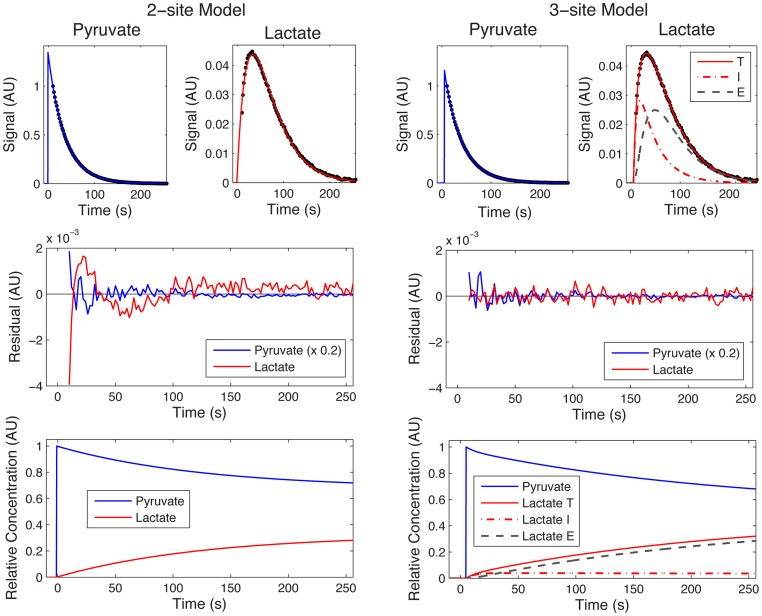
Representative dynamic spectra from a WM266.4 melanoma cell suspension. Kinetic modeling was performed using a 2-site (left) and 3-site (right) model. Total (T), intracellular (I) and extracellular (E) [1-^13^C]lactate fits, derived from the 3-site kinetic model are shown. Residuals between the data and the model are shown (central row). The concentration curves (bottom) were generated by correcting data for hyperpolarized relaxation.

Figure 3 shows time-curves of the integrals of the dynamic spectra acquired from WM266 melanoma cells, and are a representative example of the *in vitro* data used in this study. Hyperpolarized [1-^13^C]pyruvate and [1-^13^C]lactate spectra were fitted according to a 2-site or a 3-site kinetic model using a modified cost function (see Text S1). The lactate residual from the 2-site model fit shows that some structure remains, and both pyruvate and lactate residuals from the 2-site model fit are significantly larger than from the 3-site model fit. Also shown are the relative concentrations of pyruvate and lactate, estimated by correcting the hyperpolarized signals for relaxation, using effective relaxation rate constants derived from the model fitting. In the 3-site model, intracellular and extracellular lactate effective relaxation rates were assumed to be the same [19]. Figure 3 shows that intracellular lactate levels reach a steady state within approximately 10 seconds, and levels were significantly lower than extracellular lactate.

**Figure 4:**
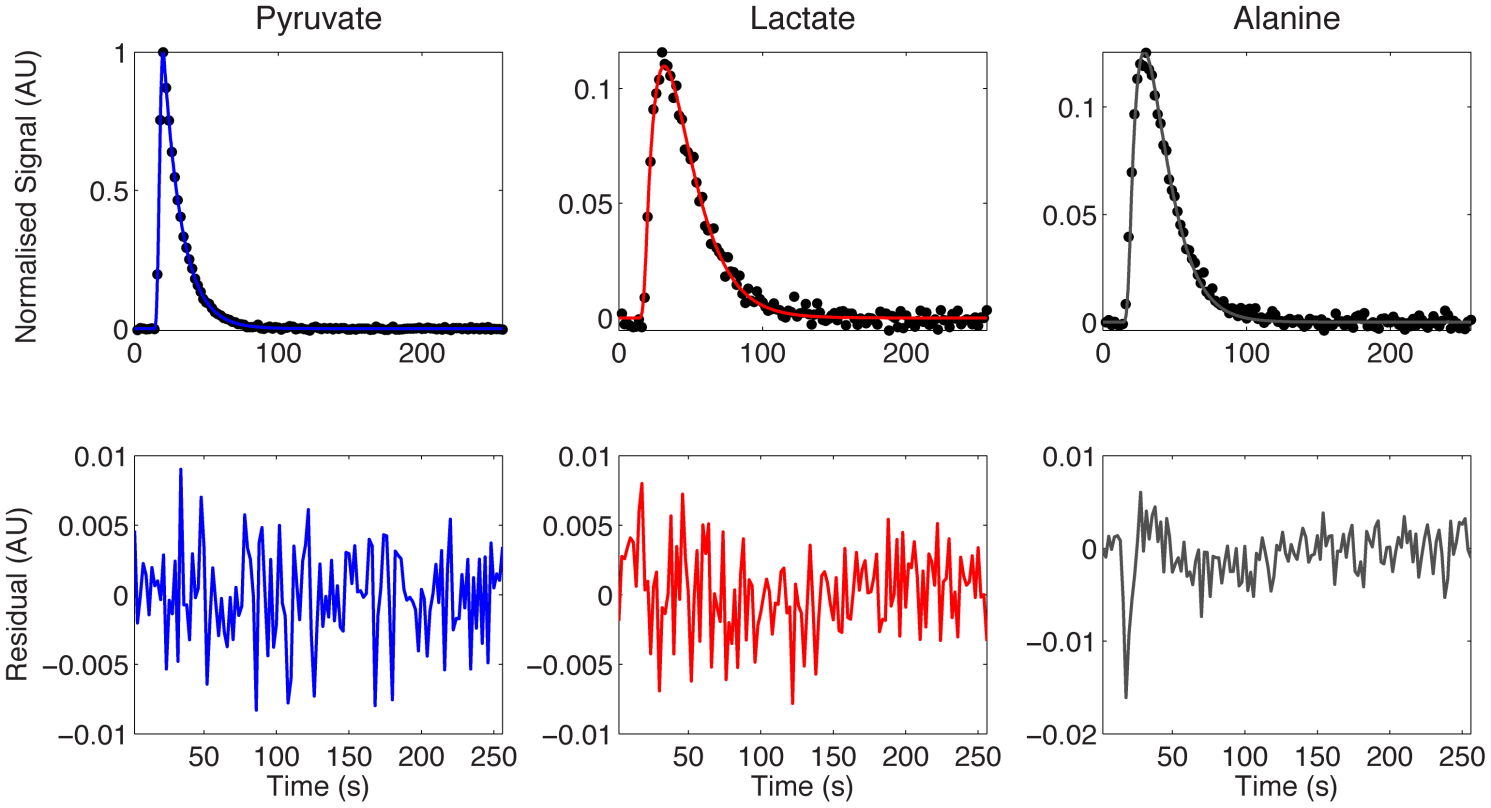
Representative dynamic ^13^C time-courses from an *in vivo* HT29 colon carcinoma xenograft at 7 T. The model fits to pyruvate, lactate and alanine are shown with a solid line and residuals between the data and the plots are displayed.

Figure 4 shows representative integrals of the dynamic MRS spectra from an HT29 colon carcinoma xenograft at 7 T for the pyruvate, lactate and alanine peaks. Inset on the pyruvate curve is the derived plasma curve calculated from the fitted parameters. The 2-site model fitting showed random unstructured residuals.

**Figure 5:**
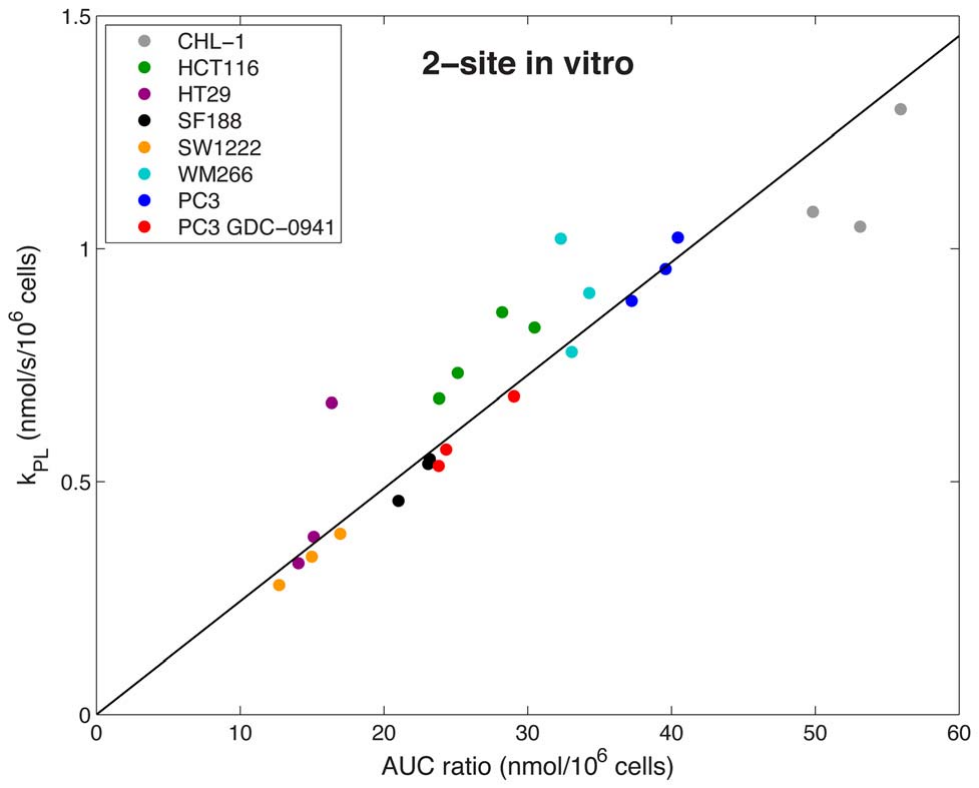
*In vitro* AUC ratios plotted against forward rate constant (*k_PL_*), derived from the 2-site model. Data is normalized to initial pyruvate concentration and cell number. An excellent correlation is observed between AUC ratio and *k_PL_* across a range of cell lines. Clustering between cell types can also be seen, and spread between data points of the same cell type tends to be in the direction of the best-fit line.

**Figure 6:**
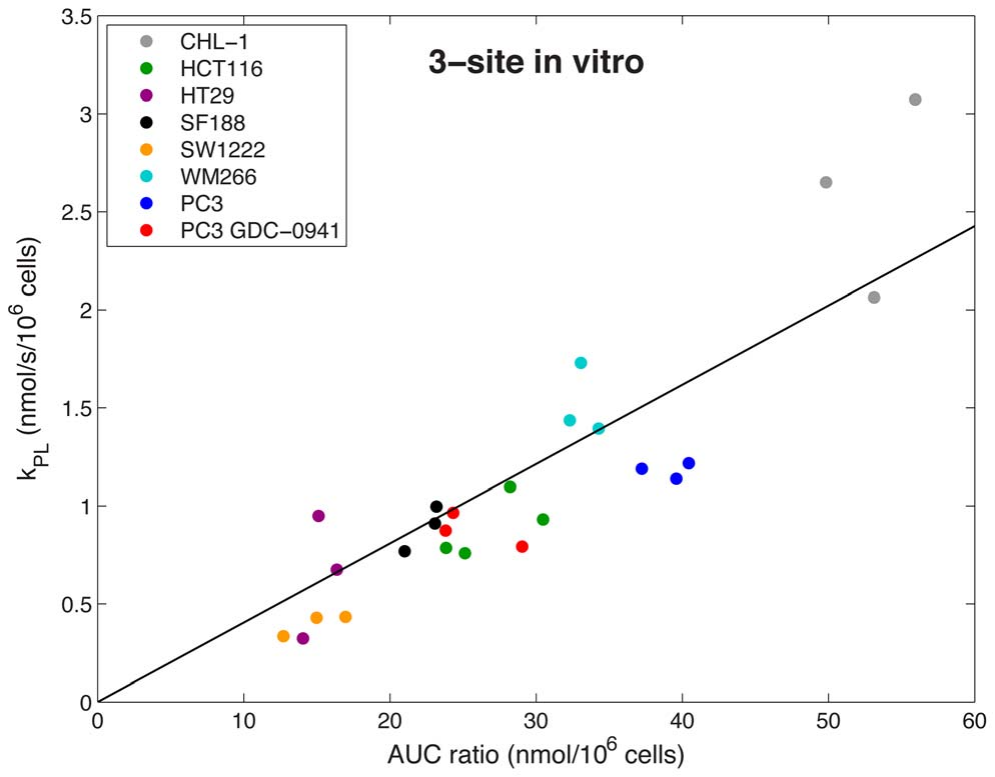
*In vitro* AUC ratios plotted against forward rate constant (*k_PL_*), derived from the 3-site model. Data is normalized to initial pyruvate concentration and cell number. A strong correlation is observed between AUC ratio and *k_PL_* across a range of cell lines. Clustering between cell types can also be seen.

Figures 5 and 6 show the relationship between the AUC ratio (total ^13^C lactate/total ^13^C pyruvate) and the apparent forward rate constant (*k_PL_*) for *in vitro* data derived from the 2-site model and the 3-site model respectively. Data are normalized to initial pyruvate concentration and cell number (per million cells). *In vitro k_PL_* values are consistent with those previously reported for various cancer cell lines [9], [24]–[26]. Both models show a very good correlation between AUC ratio and *k_PL_*, where the Pearson correlation coefficient (r) and p-value were as follows: 2-site, r = 0.921, p<0.0001; 3-site, r = 0.882, p<0.0001 (statistics derived assuming linear fit through zero). In almost all cases, inclusion of MCT transport of lactate out of the cell in the 3-site model gives higher estimates of *k_PL_*, which is in agreement with previous observations [19]. The slopes of the lines are 0.0243±0.0007≈*r_L_*+*k_LP_* (2-site) and 0.0405±0.0020≈*r_L_*+γ*k_LP_* (3-site) again highlighting the influence of transport. Response of PC3 cells treated with the PI3K inhibitor GDC-0941 was detected using the AUC ratio, with a significant (p = 0.002, Student's unpaired, two-tailed t-test) reduction in AUC ratio between control and treated data (normalized to cell number). Significant reduction in *k_PL_* was also detected using the 2-site and 3-site models (p = 0.004 (2-site), p = 0.006 (3-site), Student's unpaired, two-tailed t-test).

**Figure 7:**
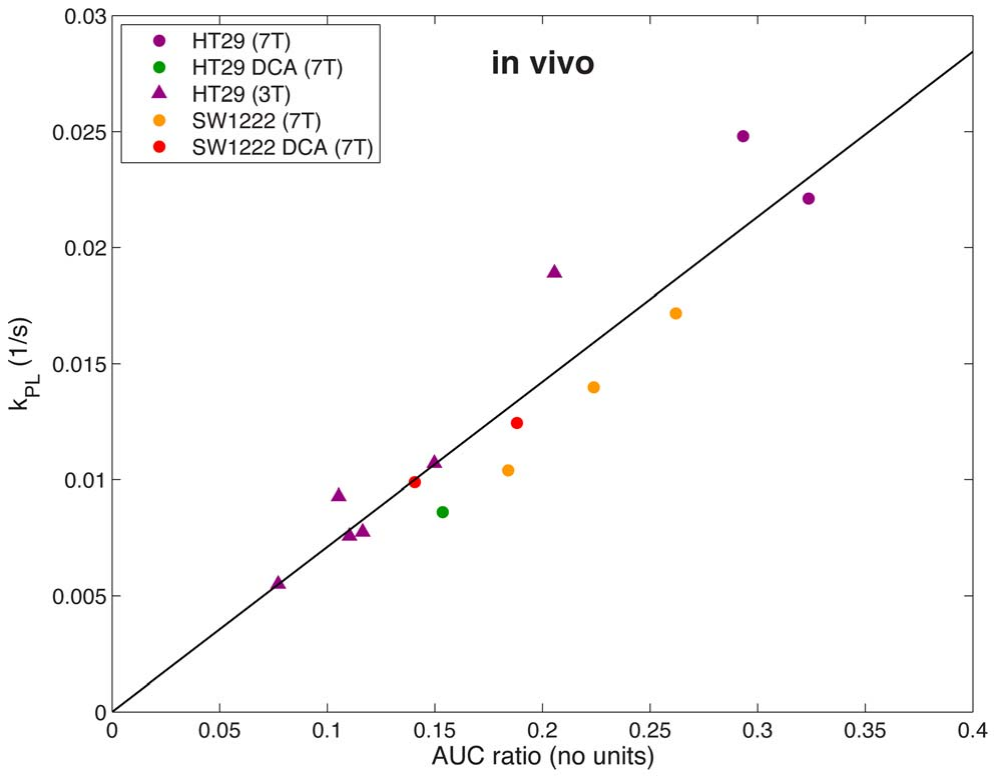
*In vivo* AUC ratios plotted against forward rate constant (*k_PL_*), derived from kinetic modeling. Data was acquired from tumor xenografts at 3 T (triangles) or 7 T (circles). A strong correlation is observed between AUC ratio and *k_PL_* at 3 T and 7 T for both HT29 and SW1222 xenografts. Drug treatment with dichloroacetate (DCA) did not appear to affect the relationship between *k_PL_* and AUC ratio.

Figure 7 shows the relationship between the AUC ratio and the forward rate constant (*k_PL_*) derived from the *in vivo* data. There is excellent correlation between the AUC ratio and *k_PL_*, with r = 0.932 and p<0.0001. The slope of the line is 0.0711±0.0029≈*r_L_*+*k_LP_*. *In vivo k_PL_* values are consistent with those previously reported for various preclinical cancer models [9], [12], [14], [27], [28]. A small number of DCA treatment data (n = 2 SW1222, n = 1 HT29) fit the trend of the control data sets, and suggest that the relation between AUC and *k_PL_* is not affected by drug treatment.

## Discussion

While the AUC ratio analysis presented here is novel, the kinetic models used throughout this work have been widely used in cancer studies and other areas. Previous studies have used the assumption that reactions follow first order rate kinetics; we have applied these assumptions in deriving the AUC ratio analysis. The quality of the correlation suggests that these assumptions are valid for exchange reactions (e.g. LDH) close to equilibrium. However, for reactions that may violate this assumption, due to, for example, limited enzyme cofactors or reactions far from equilibrium, both the modeling and AUC analysis methods would need re-evaluating.

The 2-site and 3-site *in vitro* model fits and corresponding residual plots (Figure 3) show that the lactate residual from the 2-site model is structured, which is evidence that the 2-site model does not completely describe the data. This is not the case in the 3-site model, where the lactate residuals are lower and show no evidence of structure. The concentration curves from the 3-site model show that intracellular [1-^13^C]lactate quickly reaches a steady state after injection of hyperpolarized pyruvate, and most of the hyperpolarized signal originates from outside the cell, which is contrary to a recent observation *in vivo* by Kettunen *et al.* [29]. This is an interesting result and could be investigated further by performing diffusion-weighted spectroscopy to distinguish intracellular and extracellular compartments, as suggested by Koelsh *et al.* [30]. Furthermore, characteristics of pyruvate and lactate transport by MCT1 and MCT4 have been investigated using cell perfusion systems and hyperpolarized pyruvate [24], [31]. These studies show that MCT transport rates can act as biomarkers of cellular aggressiveness and of treatment response, thus highlighting the potential advantages of the 3-site kinetic model.

*In vitro* there was a strong correlation with the AUC ratio for both 2-site and 3-site kinetic models, across a wide range of cancer cell types and in response to treatment with GDC-0941, a PI3K inhibitor, or vehicle. The slightly better correlation for the 2-site model over the 3-site model (Figures 5 and 6, respectively) may be due to larger errors on *k_PL_* estimates as a result of the increased number of parameters to be estimated in the 3-site model, or due to an increased variation in the denominator term (*r_L_*+γ*k_LP_*). The AUC ratio method detected response to GDC-0941 treatment with slightly greater significance than 2-site and 3-site kinetic models, and this reduction in AUC ratio and *k_PL_* is consistent with previous work relating to PI3K inhibition in cancer [8].

*In vivo*, preliminary DCA treatment data were included to inform whether DCA drug treatment affects the relationship identified between the AUC ratio and *k_PL_* compared with control data. While there are too few DCA treatment data points to draw any significant conclusion, we show that in these limited cases (n = 2 SW1222, n = 1 HT29), the relationship between *k_PL_* and AUC ratio also holds following drug treatment. Both *k_PL_* and AUC ratio were generally less in treated xenografts compared with controls, but a larger study is needed to confirm this. These preliminary results suggest that the AUC ratio is an appropriate method for assessing treatment response *in vivo* and *in vitro*.

An additional source of variation in the *in vitro* AUC data is that the first few data points are missing, owing to injection of [1-^13^C]pyruvate into the cell suspension outside of the NMR magnet, which is evident in the [1-^13^C]lactate curve in Figure 3. This phenomenon need not be considered in the *in vivo* data sets because the entireties of the metabolite curves are recorded. The AUC ratio for *in vitro* results is therefore affected by missing the first few data points, whereas the kinetic model is able to interpolate the curves back to t = 0. We have assessed the significance of this variation by comparing the correlation coefficients between *k_PL_* and the AUC ratios computed directly from the data, with those obtained by evaluating the formulae in equations (5) and (6) with the estimated parameters, which correct for missing data points. For the 2-site model the r-value increases from 0.921 to 0.953 with the model derived AUC ratio, and for the 3-site model from 0.882 to 0.906. While both r-values are improved with the model derived AUCs, the effect is small. This source of variation can be reduced by starting the data acquisition at a fixed time after the [1-^13^C]pyruvate is added to the cells, or where possible, starting the acquisition before the [1-^13^C]pyruvate is added so that the entire time-course is acquired. The effect on relative root-mean-square errors in estimating T_1_ and *k_PL_* from kinetic modeling of simulated data was also investigated by Santarelli *et al.* and they concluded that, while the inclusion of the whole data set is beneficial, only very small errors are incurred through truncation of the first few data points [15]. Another alternative is a simple correction scheme that assumes the missing lactate and pyruvate signals vary approximately linearly with time, although we do not explore this here.

The slope of the *in vivo* best-fit line (Figure 7) is two times greater than the slope of the *in vitro* 2-site data (Figure 5), which can be attributed to the different physiological environments that the reactions are occurring in, affecting *r_L_* and *k_LP_*. *In vivo* hyperpolarized relaxation rates are greater, and reaction kinetics are affected by substrate delivery and surrounding tissues, which do not influence the *in vitro* reaction. Due to the complexity of these additional factors in the *in vivo* experiment and the poorer signal-to-noise compared to the *in vitro* data we did not apply a 3-site model in this case. The significant alanine signal observed *in vivo* but not *in vitro* (Figure 2) can be explained by the absence of a sufficient alanine pool *in vitro* for the pyruvate–alanine exchange reaction to take place. The *in vitro* reaction could also be limited by glutamate availability. Additionally, the signals detected *in vivo* could be influenced by the transport of metabolic products from surrounding tissues into the region of interest by the animal's circulatory system.

There are several advantages of using the AUC ratio over kinetic modeling as a means to compare hyperpolarized data sets. The AUC ratio provides a robust and simple method to quantify hyperpolarized data with equal, if not greater sensitivity to detecting therapeutic response. The AUC ratio, unlike kinetic modeling, is independent of the input function for the injected metabolite, which is particularly relevant for clinical applications where measuring the AIF is challenging or not feasible. An alternative model-free approach was presented in [32] where the ratios of maximum lactate/pyruvate signals were used to detect response to treatment *in vivo*. In the AUC ratio method proposed here, utilization of the entire data set generates a metric with lower associated error than using the maximum-recorded signal alone, and in addition, a mathematical analysis relating the AUC ratio to the underlying kinetics can be performed.

The AUC ratio can be calculated by adding together the peak areas of each spectrum from the dynamic series as shown in Eq. (7), or equivalently by summing the spectra before calculating the peak area. In the case of low concentration metabolites, while the peaks in the individual spectra may be below the noise floor, the corresponding peak in the averaged (summed) spectra is likely to be visible due to the noise reduction of the averaging process. This improves the signal-to-noise ratio (SNR) in the averaged spectrum, which enables metabolites with slow kinetics and/or low signals to be detected, and their AUC ratios to be quantified.

## Conclusions

We have shown that a model-free method for quantification of hyperpolarized dynamic spectra based on simple AUC ratios yields a metric that is proportional to the rate constant of the reaction and have validated the approach using *in vitro* and *in vivo* data at different field strengths. We further show that the methodology is valid *in vitro* for both 2-site and 3-site kinetic models by comparing *k_PL_* derived from kinetic modeling with the AUC ratio. An excellent correlation was found between AUC ratio and *k_PL_* in all cases. The AUC method can potentially be used to quantify low concentration metabolites, where SNR is insufficient for kinetic modeling. To do this, the sum of all individual spectra can be taken to improve the metabolite SNR before calculating the AUC. Furthermore, we have shown that the AUC ratio is independent of the AIF for the injected hyperpolarized metabolite and is applicable for cases where multiple hyperpolarized metabolites are formed from the injected substrate. In conclusion, this model-free method provides a robust and clinically relevant approach for the analysis of hyperpolarized spectra, where measurement of the input function is problematic.

## Supporting Information

Text S1 Mathematical derivations including three sections: Accounting for signal loss due to RF excitations, Laplace transforms for area under the curve ratio calculations, Modified cost function for kinetic model fitting.
(PDF)
