# Supplementary material for: Model Free Approach to Kinetic Analysis of Real-Time Hyperpolarized 13C Magnetic Resonance Spectroscopy Data
Source: PLoS One. 2013 Sep 4;8(9):e71996. doi: 10.1371/journal.pone.0071996 (PMC3762840; doi:10.1371/journal.pone.0071996)
Supplement: Text S1 — Mathematical derivations including three sections: Accounting for signal loss due to RF excitations, Laplace transforms for area under the curve ratio calculations, Modified cost function for kinetic model fitting. (PDF) [file pone.0071996.s001.pdf]

Supporting Information

Hill et al. Model Free Approach to Kinetic Analysis of Real-Time Hyperpolarized ^{13}C Magnetic Resonance Spectroscopy Data

SI Text

This supporting information provides details of a correction scheme to account for signal loss due to RF excitations, derivations of area under the curve ratios for two systems, and details of a modified cost function that is used to account for the large decaying residuals observed at the start of the in-vitro pyruvate signals.

Accounting for signal loss due to RF excitations

In this section we derive a general result that demonstrates that the signal loss incurred from each RF excitation can be accounted for by adding a constant term to the relaxation rate constants that depends on the flip angle and repetition time.

In the absence of any RF excitations, the continuous-time differential equation governing the evolution of the longitudinal magnetization of multiple metabolites is

$$\frac{\mathrm{d}\mathbf{M}}{\mathrm{d}t} = (K - R)\mathbf{M}(t) \tag{S1}$$

where the components of $\mathbf{M}(t)$ are the longitudinal magnetizations of each metabolite, K is a matrix of effective rate constants for the metabolic reactions and R is a diagonal matrix of T_1^{-1} values for each metabolite. With a repetition time of TR, RF excitations occur at times $t_n = n\cdot\mathrm{TR}$, and with a flip-angle of θ we have $\mathbf{M}(t_n^+) = \mathbf{M}(t_n^-)\cos\theta$ where t_n^+ and t_n^- indicate times just before and just after t_n. For $t_n^+ < t < t_{n+1}^-$, $\mathbf{M}(t)$ evolves according to (S1), that is

$$\mathbf{M}(t) = \exp\Big((K-R)(t - t_n^+)\Big)\cdot\mathbf{M}(t_n^+) \tag{S2}$$

Define a relaxation rate adjustment factor $r_\theta = -\mathrm{TR}^{-1}\ln(\cos\theta)$, then the magnetization just before excitation $n+1$ is

$$\begin{aligned}
\mathbf{M}(t_{n+1}^-) &= \exp\Big((K-R)(t_{n+1}^- - t_n^+)\Big)\cdot\mathbf{M}(t_n^-)\cos\theta \\
&= \exp\Big((K-R)\cdot\mathrm{TR}\Big)\cdot\mathbf{M}(t_n^-)\exp(-r_\theta\mathrm{TR}) \\
&= \exp\Big((K-R-r_\theta I)\cdot\mathrm{TR}\Big)\cdot\mathbf{M}(t_n^-)
\end{aligned} \tag{S3}$$

where I is the identity matrix. The transverse magnetization resulting from excitation n is $\mathbf{M}(t_n^-)\sin\theta$, the components of which are proportional to the measured metabolite peak areas. This combined discrete-continuous system is therefore equivalent to a purely continuous-time model with overall rate matrix $\mathcal{L} = K - R - r_\theta I$ which is sampled at times $n\cdot\mathrm{TR}$. Effective longitudinal relaxation rate constants can be defined as

$$r_i = T_{1i}^{-1} + r_\theta \tag{S4}$$

for metabolite i, then $\mathcal{L} = K - \mathrm{diag}(r_1, r_2, \ldots, r_N)$ with N metabolites.

Laplace transforms for area under the curve ratio calculations

The Laplace transform of $x(t)$ is defined as

$$\overline{x}(s) = \int_0^\infty \mathrm{e}^{-st}x(t)\,\mathrm{d}t \tag{S5}$$

and so the area under the curve (AUC) of $x(t)$ is obtained by setting $s = 0$ since

$$\overline{x}(0) = \int_0^\infty x(t)\,\mathrm{d}t. \tag{S6}$$

This identity can be used to derive AUC expressions for systems involving multiple metabolites as follows.

With first-order exchange kinetics and longitudinal relaxation the system evolves according to the following differential equation

$$\frac{\mathrm{d}\mathbf{M}}{\mathrm{d}t} = \mathcal{L}\,\mathbf{M}(t) + \mathbf{M}_{\mathrm{in}}(t) \tag{S7}$$

where $\mathbf{M}(t)$ is a vector of the time curves for multiple metabolites, $\mathcal{L}$ is the overall rate matrix and $\mathbf{M}_{\mathrm{in}}(t)$ is a vector of the metabolite input curves. Taking Laplace transforms gives

$$s\overline{\mathbf{M}}(s) - \mathbf{M}(0) = \mathcal{L}\,\overline{\mathbf{M}}(s) + \overline{\mathbf{M}}_{\mathrm{in}}(s). \tag{S8}$$

Since there are no hyperpolarized metabolites present at $t = 0$ we have $\mathbf{M}(0) = 0$ and the above expression can be re-arranged to give

$$\overline{\mathbf{M}}(s) = (sI - \mathcal{L})^{-1}\overline{\mathbf{M}}_{\mathrm{in}}(s) \tag{S9}$$

where I is the identity matrix. Setting $s = 0$ will give a vector of metabolite AUCs,

$$\overline{\mathbf{M}}(0) = -\mathcal{L}^{-1}\overline{\mathbf{M}}_{\mathrm{in}}(0). \tag{S10}$$

In our application, hyperpolarized pyruvate is the only input metabolite so

$$\overline{\mathbf{M}}_{\mathrm{in}}(0) = \begin{bmatrix} \overline{P}_{\mathrm{in}}(0) \\ 0 \\ \vdots \\ 0 \end{bmatrix} \tag{S11}$$

where $\overline{P}_{\mathrm{in}}(0)$ is the AUC of the pyruvate input curve. The AUC expression therefore simplifies to

$$\overline{\mathbf{M}}(0) = -\overline{P}_{\mathrm{in}}(0) \cdot \mathbf{v} \tag{S12}$$

where $\mathbf{v}$ is the left-most column of $\mathcal{L}^{-1}$. When AUC ratios are derived from the elements of $\overline{\mathbf{M}}(0)$ the $\overline{P}_{\mathrm{in}}(0)$ terms cancel, and so we obtain the key general result that when the input term contains only one metabolite, AUC ratios do not depend on the time course of the input.

Example 1: Two-site plus-one Model The two-site model [1] considers pyruvate and lactate to be purely intra-cellular, and we augment these with a third metabolite X (e.g. alanine, bicarbonate, etc.) that is in exchange with pyruvate only. The metabolite vector and rate constant matrix are

$$\mathbf{M}(t) = \begin{bmatrix} P(t) \\ L(t) \\ X(t) \end{bmatrix} \tag{S13}$$

and

$$\mathcal{L} = \begin{bmatrix} -r_P - k_{PL} - k_{PX} & k_{LP} & k_{XP} \\ k_{PL} & -r_L - k_{LP} & 0 \\ k_{PX} & 0 & -r_X - k_{XP} \end{bmatrix} \tag{S14}$$

where r_i is the effective longitudinal relaxation rate of metabolite i (see Eq. (S4)), and k_{ij} is the reaction rate constant from i to j. For the above rate matrix we obtain

$$\mathbf{v} = \frac{1}{|\mathcal{L}|} \begin{bmatrix} (r_L + k_{LP})(r_X + k_{XP}) \\ k_{PL}(r_X + k_{XP}) \\ k_{PX}(r_L + k_{LP}) \end{bmatrix} \tag{S15}$$

where $|\mathcal{L}|$ is the determinant of $\mathcal{L}$, and so

$$\begin{bmatrix} \overline{P}(0) \\ \overline{L}(0) \\ \overline{X}(0) \end{bmatrix} = -\frac{\overline{P}_{\mathrm{in}}(0)}{|\mathcal{L}|} \begin{bmatrix} (r_L + k_{LP})(r_X + k_{XP}) \\ k_{PL}(r_X + k_{XP}) \\ k_{PX}(r_L + k_{LP}) \end{bmatrix} \tag{S16}$$

The lactate to pyruvate AUC ratio is then simply

$$\frac{\overline{L}(0)}{\overline{P}(0)} = \frac{k_{PL}}{r_L + k_{LP}} \tag{S17}$$

which is proportional to the forwards rate constant k_{PL} and is independent of both $\overline{P}_{\mathrm{in}}(0)$ and the rates associated with the additional metabolite X. An equivalent result for metabolite X also holds

$$\frac{\overline{X}(0)}{\overline{P}(0)} = \frac{k_{PX}}{r_X + k_{XP}}. \tag{S18}$$

Example 2: Three-site plus-one Model The three-site model in [2] considers intra- and extra-cellular lactate separately while pyruvate remains purely intra-cellular. We augment this with a fourth metabolite X that is in exchange with pyruvate only. The metabolite vector and rate constant matrix are

$$\mathbf{M}(t) = \begin{bmatrix} P(t) \\ L_i(t) \\ L_e(t) \\ X(t) \end{bmatrix} \tag{S19}$$

and

$$\mathcal{L} = \begin{bmatrix} -r_P - k_{PL} - k_{PX} & k_{LP} & 0 & k_{XP} \\ k_{PL} & -r_L - k_{LP} - k_{LE} & k_{EL} & 0 \\ 0 & k_{LE} & -r_L - k_{EL} & 0 \\ k_{PX} & 0 & 0 & -r_X - k_{XP} \end{bmatrix} \tag{S20}$$

where k_{EL} and k_{LE} are the lactate transporter rates out of and into the cell respectively. Similar calculations as above give

$$\begin{bmatrix} \overline{P}(0) \\ \overline{L}_i(0) \\ \overline{L}_e(0) \\ \overline{X}(0) \end{bmatrix} = -\frac{\overline{P}_{\text{in}}(0)}{|\mathcal{L}|} \begin{bmatrix} \big(k_{LP}(k_{EL} + r_L) + r_L(k_{LE} + k_{EL} + r_L)\big)(r_X + k_{XP}) \\ k_{PL}(r_L + k_{EL})(r_X + k_{XP}) \\ k_{PL}k_{LE}(r_X + k_{XP}) \\ \big(k_{LP}(k_{EL} + r_L) + r_L(k_{LE} + k_{EL} + r_L)\big)k_{PX} \end{bmatrix} \tag{S21}$$

The total lactate to pyruvate AUC ratio is then

$$\frac{\overline{L}_i(0) + \overline{L}_e(0)}{\overline{P}(0)} = \frac{k_{PL}}{r_L + \gamma \cdot k_{LP}} \tag{S22}$$

where $\gamma = (k_{EL} + r_L)(k_{LE} + k_{EL} + r_L)^{-1}$. This AUC ratio is also proportional to k_{PL} and is independent of the input and additional metabolite rates. With no lactate transport from the cells $k_{LE} = 0$, which gives $\gamma = 1$ and the AUC ratio simplifies to the two-site solution, as expected.

Modified cost function for kinetic model fitting

Although the standard deviation (STD) of the lactate residual is constant during each acquisition, the pyruvate residual starts with a STD around 20 times higher, which decays over around 100 seconds to the same level as the lactate STD. This has a biasing effect on the fitting, as the least-squares (LSQ) cost function is disproportionately influenced by the early pyruvate data with large residuals. We have developed a Bayesian noise model to counteract this bias that leads to a modified cost function that is approximately quadratic for pyruvate residuals of a similar magnitude to the lactate residuals, but rises more slowly than a quadratic for larger residuals.

The lactate signal model is

$$L_n = L(t_n, \mathbf{x}) + \epsilon_n \tag{S23}$$

where L_n is the nth lactate datum measured at time t_n, $L(t_n, \mathbf{x})$ is the lactate model function evaluated with rate constants in the variable $\mathbf{x}$ and ϵ_n is a sample from a Gaussian distribution with (constant) variance σ^2. Similarly, the pyruvate signal model is

$$P_n = P(t_n, \mathbf{x}) + \nu_n \tag{S24}$$

where ν_n is a sample from a Gaussian distribution with variance σ_n^2, i.e. each datum has a distinct variance. These error models lead to data likelihood functions of the form

$$p(L_n \mid \mathbf{x}, \sigma) = \frac{1}{\sqrt{2\pi\sigma^2}} \exp\left(-\frac{\big(L_n - L(t_n, \mathbf{x})\big)^2}{2\sigma^2}\right) \tag{S25}$$

$$p(P_n \mid \mathbf{x}, \sigma) = \frac{1}{\sqrt{2\pi\sigma_n^2}} \exp\left(-\frac{\big(P_n - P(t_n, \mathbf{x})\big)^2}{2\sigma_n^2}\right) \tag{S26}$$

where the distinct pyruvate variances are explicit. A prior distribution is used to model uncertainty in σ_n conditional on σ,

$$p(\sigma_n \mid \sigma) = \begin{cases} \dfrac{\sigma}{\sigma_n^2} & \sigma_n < \sigma \\ 0 & 0 < \sigma_n < \sigma \end{cases} \tag{S27}$$

which is a truncated Jeffrey's prior, as suggested in [3]. This prior encodes the constraint that $\sigma_n > \sigma$, i.e. the pyruvate residuals tend to be larger than the lactate residuals, but the prior is otherwise uninformative. Uncertainty in σ_n can be accounted for by forming the joint probability of P_n and σ_n then marginalizing σ_n via the following integral

$$\begin{aligned} p(P_n \mid \mathbf{x}, \sigma) &= \int_{\sigma}^{\infty} p(P_n \mid \mathbf{x}, \sigma_n) p(\sigma_n \mid \sigma) \, \mathrm{d}\sigma_n \\ &= \frac{\sigma}{\sqrt{2\pi}} \frac{1 - \exp\left(-\frac{\left(P_n - P(t_n, \mathbf{x})\right)^2}{2\sigma^2}\right)}{\left(P_n - P(t_n, \mathbf{x})\right)^{\mathbf{2}}} \end{aligned} \tag{S28}$$

Assuming the errors are independent, the complete likelihood function is given by the product

$$p(L_{1:N}, P_{1:N} \mid \mathbf{x}, \sigma) = \prod_{i=1}^{N} p(L_n \mid \mathbf{x}, \sigma) p(P_n \mid \mathbf{x}, \sigma) \tag{S29}$$

and the modified cost function is the negative log of this,

$$\Phi(\mathbf{x}, \sigma) = \sum_{n=1}^{N} \frac{\left(L_n - L(t_n, \mathbf{x})\right)^2}{2\sigma^2} - \ln\left(1 - \exp\left(-\frac{\left(P_n - P(t_n, \mathbf{x})\right)^2}{2\sigma^2}\right)\right) + \ln\left(\left(P_n - P(t_n, \mathbf{x})\right)^2\right) \tag{S30}$$

where any constant terms have been neglected. This function is minimized with respect to $\mathbf{x}$ and σ to yield kinetic parameter estimates that are robust to the relatively large initial pyruvate residuals.

References

1. Day SE, Kettunen MI, Gallagher FA, Hu DE, Lerche M, et al. (2007) Detecting tumor response to treatment using hyperpolarized 13C magnetic resonance imaging and spectroscopy. Nat Med 13: 1382–1387.

2. Harrison C, Yang C, Jindal A, DeBerardinis RJ, Hooshyar MA, et al. (2012) Comparison of kinetic models for analysis of pyruvate-to-lactate exchange by hyperpolarized 13 C NMR. NMR Biomed 25: 1286–1294.

3. Sivia D, Skilling J (2006) Data Analysis: A Bayesian Tutorial. Oxford: OUP. pp. 168–171.
